# Comparative effectiveness of hyperthermic intraperitoneal chemotherapy for gastric cancer: A systematic review and network meta-analysis protocol

**DOI:** 10.1097/MD.0000000000011949

**Published:** 2018-08-17

**Authors:** Zhaolun Cai, Zhaohui Cai, Tao He, Zhou Zhao, Yuan Yin, Chaoyong Shen, Xiaonan Yin, Zhixin Chen, Cao Dan, Bo Zhang

**Affiliations:** a Department of Gastrointestinal Surgery, West China Hospital, Sichuan University, Chengdu, Sichuan; b Department of Infectious Disease, Jiangsu Province Hospital of Traditional Chinese Medicine, Affiliated Hospital of Nanjing University of Chinese Medicine; c The First College of Clinical Medicine, Nanjing University of Chinese Medicine, Nanjing; d Department of Breast Surgery, West China Hospital; e Department of Medical Oncology, Cancer Center of West China Hospital, Sichuan University, Chengdu, China.

**Keywords:** gastric cancer, hyperthermic intraperitoneal chemotherapy, network meta-analysis

## Abstract

**Background:**

Comparative efficacy and safety of different hyperthermic intraperitoneal chemotherapies (HIPEC) in patients with advanced gastric cancer who underwent gastrectomy is unclear. To investigate this question, we conduct a systematic review and network meta-analysis.

**Methods:**

The protocol followed Preferred Reporting Items for Systematic Reviews and Meta-Analyses Protocols. PubMed, Embase, and the Cochrane Library will be searched systematically for eligible randomized controlled trials without language restriction. The primary outcome is overall survival. The second outcomes are postoperative complications. The surface under the cumulative ranking curve value will be calculated to establish a hierarchy of the treatments.

**Results:**

The results will provide useful information about the effectiveness and safety of HIPEC regimens in patients with resected gastric cancer.

**Conclusion:**

The findings of the study will be disseminated through peer-reviewed journal.

## 1 Introduction

Stomach cancer is the third leading cause of death from cancer worldwide in 2016.^[1]^ Complete surgery remains the major curative treatment for localized gastric cancer.^[2,3]^ Even despite potentially curative surgery, the prognosis of the patients with the disease remains poor mainly due to peritoneal recurrence or local recurrence after radical gastrectomy.^[4–6]^ Peritoneal recurrence was observed most frequently in recurrent gastric cancer.^[6]^ The high risk of peritoneal recurrence prompted the investigation of hyperthermic intraperitoneal chemotherapy (HIPEC) as a therapeutic option, and the efficacy of which has been confirmed by previous studies.^[7–10]^ However, there is no established consensus on optimal HIPEC regimens because of the shortage of head-to-head trials and the limitation of traditional pair-wise meta-analyses. As the novel treatments are being developed to improve the survival of patients with advanced gastric cancer, the efficacy and safety of HIPEC regimens require closer and more sophisticated evaluation.

In this study, we will perform a systematic review and network meta-analysis to investigate the comparative effectiveness and safety of enrolled HIPEC regimens in patients with advanced gastric cancer who underwent gastrectomy.

## 2 Methods

The protocol follows PRISMA-P checklist,^[11]^ and the study will follow PRISMA guidelines.^[12]^ The systematic review and meta-analysis will be conducted following an established protocol (PROSPERO: CRD42018099451). The study is a meta-analysis of aggregate data which do not involve human subjects and do not need ethical approval.

### 2.1 Eligibility criteria

The detailed eligibility criteria are summarized using PICOS approach (patients, intervention, comparisons, outcome, and study design type).

#### 2.1.1 Patients and comparison of interventions

We will include studies that contain patients with gastric cancer treated with at least 2 arms of following treatments: surgery with different HIPEC regimens or surgery alone. Studies should provide sufficient data of survival rates. There are no restrictions in age, ethnic distribution, and gender.

#### 2.1.2 Outcomes

The primary outcome is overall survival (OS). The second outcome is postoperative morbidity.

#### 2.1.3 Study design

Published RCTs with no language restriction will be included in the meta-analysis.

### 2.2 Information sources

PubMed, Embase, and the Cochrane library (Jun 2018) will be systematically searched for eligible studies.

### 2.3 Search strategy

Search strategy of PubMed was as follows:

#1 (((((Gastric cancer) OR stomach cancer) OR stomach neoplasm) OR “Gastric cancers”) OR “stomach cancers”) OR “stomach neoplasms”
#2 (((intraperitoneal perfusion) OR “peritoneal perfusion”) OR hyperthermic intraperitoneal chemotherapy) OR intraperitoneal chemotherapy
#3 ((((((((“Randomized Controlled Trial” [Publication Type]) OR “Controlled Clinical Trial” [Publication Type]) OR “randomized” [tiab]) OR “placebo” [tiab]) OR “Clinical Trials as Topic”[Mesh:NoExp]) OR “randomly” [tiab]) OR “trial” [ti])) NOT ((“Animals” [mh]) NOT “ humans” [mh])
#4 #1 AND #2 AND #3

### 2.4 Study selection and data extraction

**Figure 1:**
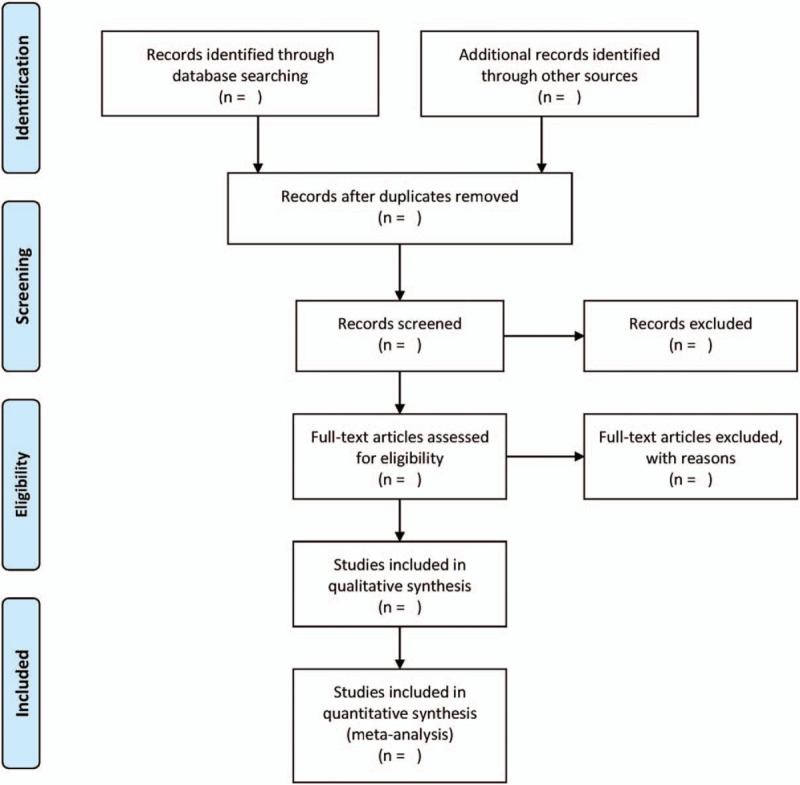
PRISMA flow diagram.

Two reviewers will perform study selection and data extraction. The selection process will be summarized in a PRISMA flow diagram (Fig. 1). The data include study characteristics (authors, year of publication, and countries), data needed for quality assessment, and characteristics of patients including intervention, follow-up time, mean age, outcomes and tumor pathologic variables. Hazard ratios (HRs) will be extracted from studies on the basis of reported values or be estimated from survival curves by established methods.^[13]^ All study characteristics will be summarized in the same standardized collection form by 2 reviewers.

### 2.5 Risk of bias

The risk of bias of included RCTs will be independently assessed by 2 investigators using the Cochrane Collaboration's tool for assessing risk of bias in randomized trials^[14]^ in terms of sequence generation, allocation concealment, blinding of participants and researchers, incomplete outcome data, selective reporting, and other bias random. Disagreements will be resolved in group discussion.

### 2.6 Data synthesis and statistical analysis

#### 2.6.1 Pairwise meta-analyses

R software version 3.5.0 will be used to perform pairwise meta-analyses. Pooled odds ratio (OR) will be calculated for dichotomous data. Pooled HR will be calculated for time-to-event data.

We will measure the heterogeneity of the included studies by *I*^2^ statistic. χ^2^ test with the significance set *P < *.10 or I2 > 50% indicates statistical heterogeneity.^[15]^ A fixed effect will be used to calculate the outcomes when statistical heterogeneity is absent, whereas a random-effects model will be used. Publication bias will be assessed by Begg's funnel plot and Egger regression, if the study includes 10 or more studies.^[16,17]^

#### 2.6.2 Network meta-analyses

A Bayesian network meta-analysis will be performed with R x64 3.5.0. We will use the node splitting method to assess the inconsistency between direct and indirect comparisons if a loop exists.^[18]^ Surface under the cumulative ranking area (SUCRA) values will be used to rank the different HIPEC regimens.^[19]^ Comparison-adjusted funnel plots will be drawn to detect the small sample effects of on the results. A network plot will be conducted to present the comparisons of the treatment across trials to ensure if a network meta-analysis is feasible. Studies will be excluded if the treatments investigated are not connected by other treatments. All the result figures will be generated using R x64 3.5.0 and STATA version 14.0 (College Station, TX).

## 3 Discussion

Currently, the optimal HIPEC regimen for resected gastric cancer is still uncertain. Therefore, we conduct a network meta-analysis to investigate the question. To the best of our knowledge, this is the first network meta-analysis in the area. We aim to summarize direct and indirect evidence and provide evidence-based suggestions for the clinical use of HIPEC.

## Author contributions

Bo Zhang, Dan Cao and Zhaolun Cai conceived the concept and designed the study protocol. Zhaohui Cai and Tao He tested the feasibility of the study. Zhixin Chen, Chaoyong Shen, Yuan Yin, Xiaonan Yin, and Zhou Zhao wrote the manuscript. Bo Zhang, Dan Cao and Zhaolun Cai provided methodological advice, polished and revised the manuscript. All authors saw and approved the final version of the paper.

**Conceptualization:** Zhaolun Cai, Cao Dan, Bo Zhang.

**Investigation:** Zhaohui Cai, Tao He.

**Methodology:** Zhaolun Cai, Cao Dan, Bo Zhang.

**Writing – original draft:** Zhou Zhao, Yuan Yin, Chaoyong Shen, Xiaonan Yin, Zhixin Chen.

**Writing – review & editing:** Zhaolun Cai, Cao Dan, Bo Zhang.

Zhaolun Cai: 0000-0002-3706-6703
