# Comparative effectiveness of hyperthermic intraperitoneal chemotherapy for gastric cancer: A systematic review and network meta-analysis protocol: Erratum

**DOI:** 10.1097/MD.0000000000014487

**Published:** 2019-02-08

In the article, “Comparative effectiveness of hyperthermic intraperitoneal chemotherapy for gastric cancer: A systematic review and network meta-analysis protocol”,^[1]^ which appeared in Volume 97, Issue 33 of *Medicine*, Dr. Dan Cao's name appeared incorrectly throughout the article as Cao Dan.

The corresponding author information should be “Correspondence: Bo Zhang, Department of Gastrointestinal Surgery, West China Hospital, Sichuan University, Chengdu 610041, Sichuan, China (e-mail: hxwcwk@126.com); Dan Cao, Department of Medical Oncology, Cancer Center of West China Hospital, Sichuan University, Chengdu 610041, China (e-mail: caodan316@163.com).”
